# Homocysteine levels, genetic background, and cognitive impairment in Parkinson’s disease

**DOI:** 10.1007/s00415-022-11361-y

**Published:** 2022-09-28

**Authors:** María Teresa Periñán, Daniel Macías-García, Silvia Jesús, Juan Francisco Martín-Rodríguez, Laura Muñoz-Delgado, Maria Valle Jimenez-Jaraba, Dolores Buiza-Rueda, Marta Bonilla-Toribio, Astrid Daniela Adarmes-Gómez, Pilar Gómez-Garre, Pablo Mir

**Affiliations:** 1 Servicio de Neurología y Neurofisiología Clínica, Unidad de Trastornos del Movimiento, Instituto de Biomedicina de Sevilla, (IBiS), Hospital Universitario Virgen del Rocío/CSIC/Universidad de Sevilla, Avda. Manuel Siurot s/n, 41013 Seville, Spain; 2 Centro de Investigación Biomédica en Red sobre Enfermedades Neurodegenerativas (CIBERNED), Madrid, Spain; 3 Departamento de Psicología Experimental, Facultad de Psicología, Universidad de Sevilla, Seville, Spain; 4 Departamento de Medicina, Facultad de Medicina, Universidad de Sevilla, Seville, Spain

**Keywords:** Parkinson’s disease, Homocysteine, Cognitive impairment, Meta-analysis, *MTHFR*

## Abstract

**Background:**

Hyperhomocysteinemia is considered an independent risk factor for cognitive impairment.

**Objective:**

To study the correlation between homocysteine levels and cognitive impairment in patients with PD.

**Methods:**

We conducted a case–control study that included 246 patients with PD, of whom 32 were cognitively impaired. The levels of homocysteine, folate, and vitamin B12 were measured in peripheral blood. Multivariate logistic regression analysis was applied to determine differences in homocysteine levels between PD patients with and without cognitive impairment. A meta-analysis was performed to clarify the role of Hcy levels in PD with cognitive decline. Five polymorphisms in genes involved in Hcy metabolism, including *MTHFR* rs1801133 and rs1801131, *COMT* rs4680, *MTRR* rs1801394, and *TCN2* rs1801198, were genotyped.

**Results:**

Our case–control study showed that homocysteine levels were associated with cognitive impairment in PD after adjusting for possible confounding factors such as levodopa equivalent daily dose. The results of our meta-analysis further supported the positive association between homocysteine levels and cognition in PD. We found that the *MTHFR* rs1801133 TT genotype led to higher homocysteine levels in PD patients, whereas the *MTHFR* rs1801131 CC genotype resulted in higher folate levels. However, the polymorphisms studied were not associated with cognitive impairment in PD.

**Conclusions:**

Increased homocysteine levels were a risk factor for cognitive decline in PD. However, no association was found between polymorphisms in genes involved in homocysteine metabolism and cognitive impairment in PD. Large-scale studies of ethnically diverse populations are required to definitively assess the relationship between *MTHFR* and cognitive impairment in PD.

**Supplementary Information:**

The online version contains supplementary material available at 10.1007/s00415-022-11361-y.

## Introduction

Parkinson’s disease (PD) is a progressive neurodegenerative disorder that encompasses motor, cognitive, behavioral, and autonomic features [1]. There is growing evidence that oxidative stress is involved in the pathophysiology, disease progression, and development of cognitive impairment in PD [2].

In recent years, several studies have shown that nearly 30% of PD patients have increased plasma homocysteine (Hcy) levels [3]. Hcy is an amino acid generated through the demethylation of methionine. The Hcy produced in the human body is mainly eliminated through (1) remethylation, in which Hcy is remethylated to methionine with vitamins B2 and B12 as cofactors; (2) transsulfuration, in which Hcy is first transformed into cystathionine (with vitamin B6 as cofactor) to be metabolized into cysteine and α-ketobutyric acid, which are ultimately excreted from the body; and (3) released into the extracellular fluid [4]. Elevated plasma Hcy levels might be due to impaired metabolism due to genetic variants in genes that encode enzymes involved in Hcy metabolism. Several functional polymorphisms within the methylenetetrahydrofolate reductase (*MTHFR*), methionine synthase reductase (*MTRR*), catechol-*O*-methyltransferase (*COMT*), and transcobalamin II (*TCN2*) genes have been shown to affect Hcy metabolism [5–7].

The enzymes involved in the metabolism of methionine depend on B vitamins: vitamin B12, vitamin B6, and folic acid. A deficiency of folate and vitamin B12 leads to the atrophy of CA1 neurons in the hippocampus and the disruption of cognitive processes with increased Hcy [8]. There is evidence that elevated Hcy levels are associated with a decline in cognitive functioning [9, 10]. Sampedro et al*.* described an association between higher Hcy levels with frontal cortical thinning and increased intracortical diffusivity in frontal and temporo-occipital regions. The observed microstructural alterations in these regions correlated, in turn, with cognitive performance. However, the literature on Hcy levels and cognitive impairment in PD is mixed. Some reports have found impaired cognition in PD patients with hyperhomocysteinemia [7, 11–18], while others found no relationship between Hcy levels and cognitive impairment in PD [19, 20]. The discrepancies in the literature can be attributed to the sample size, the comprehensiveness of cognitive tests, or the interindividual genetic variability.

Consequently, the present study aimed to examine whether elevated Hcy levels were associated with cognitive impairment in PD. We first investigated differences in Hcy levels between PD patients with and without cognitive impairment through a case–control study. Subsequently, we performed a meta-analysis to clarify the role of Hcy levels in PD with cognitive decline. Then, we analyzed whether single nucleotide polymorphisms (SNPs) in the *MTHFR*, *MTRR, COMT* and *TCN2* genes correlate with hyperhomocysteinemia and contribute to cognitive dysfunction in PD.

## Materials and methods

### Participants and study design

We included 246 patients with PD from the Movement Disorders Clinic of the Hospital Universitario Virgen del Rocio in Seville (Spain), diagnosed following the Movement Disorders Society (MDS) clinical diagnostic criteria for PD [21].

All subjects underwent a medical assessment by movement disorders specialists. PD patients were evaluated in the “on” motor state and good dopaminergic response. The diagnosis of cognitive impairment in PD was defined according to the MDS clinical diagnostic criteria [22, 23]. Global cognitive function was evaluated using the results of the neuropsychological assessment and the scores on standard scales (with the cut-off scores for cognitive impairment) such as the Mattis Dementia Rating Scale ($\le$ 139), Parkinson’s Disease Cognitive Rating Scale ($\le$ 81), Mini Mental State Examination (MMSE) ($\le$ 24), Montreal Cognitive Assessment ($\le$ 26), Scales for Outcomes in Parkinson’s Disease-Cognition ($\le$ 22), and Parkinson’s Disease Dementia Short Screen ($\le$ 11) [24–29] as screening tools. Consequently, we identified PD patients who met the diagnostic criteria for mild cognitive impairment or dementia in a long-term review of medical records. All patients with PD with cognitive impairment underwent brain magnetic resonance as well as biochemical analyses to exclude non-degenerative/metabolic causes of cognitive impairment. Furthermore, all participants were examined for exclusion criteria that could influence Hcy levels at the time of blood extraction or any other relevant neurological disease.

Hcy, folate, and vitamin B12 levels were measured in peripheral blood. The study was approved by the local ethics committee in accordance with the Declaration of Helsinki, and written consent was obtained from all participants prior to blood withdrawal.

### Genetics

Genomic DNA was isolated from peripheral blood samples according to established protocols using standard or automated methods (DNA Isolation Kit for Mammalian Blood, Roche Diagnostics, Indianapolis, IN, USA; MagNA Pure LC, Roche Diagnostics, Indianapolis, IN, USA). DNA quantification was determined by a NanoDrop2000 spectrophotometer (Thermo Fisher Scientific, Waltham, MA, USA).

All participants were genotyped for rs1801133 and rs1801131 (*MTHFR*), rs4680 (*COMT*), rs1801394 (*MTRR*) and rs1801198 (*TCN2*). Genotyping was performed using Taqman SNP Genotyping Assays (Applied Biosystems, Foster City, CA, USA) in a LightCycler480-II (Roche Applied Science, Penzberg, Germany).

### Statistical analysis

All analyses were performed using the statistical software R v4.0.4 and the PLINK software v1.07. Demographic and clinical variables were examined for normality using Shapiro–Wilk testing. Comparisons of means were made using independent t-test or the Kruskal–Wallis test. Chi-square test was used to assess the binary outcome variables. To examine the association between cognitive impairment and Hcy levels in PD, multivariate logistic regression analysis was used controlling for age, sex, levodopa equivalent daily dose (LEDD), disease duration, folate, and vitamin B12 levels. The significance level for all statistical tests was set at 0.05.

The association between polymorphisms and Hcy levels in PD was assessed using multivariate linear logistic regression models adjusted for sex, age, LEDD, folate, and vitamin B12 levels. Furthermore, the association of these SNPs with folate and vitamin B12 levels in PD was evaluated using multiple linear regression models adjusted for sex and age. We further assessed the association between cognitive impairment in PD and these SNPs using logistic regression analyses adjusted for sex, age, LEDD, disease duration, folate, and vitamin B12 levels. All results were corrected for multiple testing using the Bonferroni correction method. A *P* < 0.01 in the Hardy–Weinberg equilibrium test and a minor allele frequency of 1% were established as quality controls.

### Meta-analysis

We performed this meta-analysis according to the Preferred Reporting Items for Systematic Reviews and Meta-analyses (PRISMA) statement. Detailed information on meta-analysis methods can be found in the Supplementary Material.

## Results

### Observational case–control study

After applying inclusion and exclusion criteria, a total of 246 PD patients were included. Demographic and clinical data of the study participants are shown in Table 1. PD patients with cognitive impairment showed significantly higher Hcy levels compared to PD patients without cognitive decline (21.8 ± 7.9 vs. 17.5 ± 6.3 μmol/L, *P* = 0.040). We also observed that PD patients with cognitive impairment were older, they presented an older age at the onset of the disease, and showed a longer duration of the disease than PD patients without cognitive decline. Furthermore, the levodopa requirements according to LEDD were higher in patients with PD with cognitive impairment.

**Table 1:** Demographic and clinical data in PD patients with cognitive impairment and PD patients without cognitive impairment

Parameter	Total PD (*N* = 246)	PD without cognitive impairment (*N* = 214)	PD with cognitive impairment (*N* = 32)	*P* value
Sex (% men)	144 (58.5)	129 (60.3)	15 (46.9)	0.15^a^
Age (y), mean ± SD	62.7 ± 11.4	61.7 ± 11.5	69.3 ± 7.8	< 0.001^b^
Age at onset (y), mean ± SD	52.9 ± 12.3	52.3 ± 12.6	56.9 ± 9.6	0.043^c^
Disease duration (y), mean ± SD	9.9 ± 6.8	9.4 ± 6.9	12.8 ± 6.0	0.005^c^
Hcy (μmol/L), mean ± SD	18.1 ± 6.7	17.5 ± 6.3	21.8 ± 7.9	0.040^d^
Folate (ng/mL), mean ± SD	8.3 ± 4.2	8.3 ± 4.1	8.1 ± 4.7	0.821^c^
Vit B12 (pg/mL), mean ± SD	399.0 ± 200.1	396.9 ± 200.5	413.1 ± 200.5	0.672^c^
LEDD, mean ± SD	793.2 ± 479.1	766.8 ± 488.1	969.5 ± 374.5	0.009^c^

*PD* Parkinson’s disease, *N* total number of subjects, *SD* standard deviation, *Hcy* homocysteine, *Vit B12* vitamin B12, *LEDD* levodopa equivalent daily dose

^a^Based on chi-squared test

^b^Based on Kruskal–Wallis test

^c^Based on *T*-Student test

^d^Multivariate logistic regression adjusted for age, sex, LEDD, disease duration, folate and vitamin B12 levels

### Meta-analysis

After applying inclusion and exclusion criteria, 12 articles were selected for this meta-analysis. Along with our study, 13 Hcy levels mean differences were included, comprising 568 PD patients with cognitive impairment and 1241 PD patients without cognitive decline (Table S1).

**Fig. 1:**
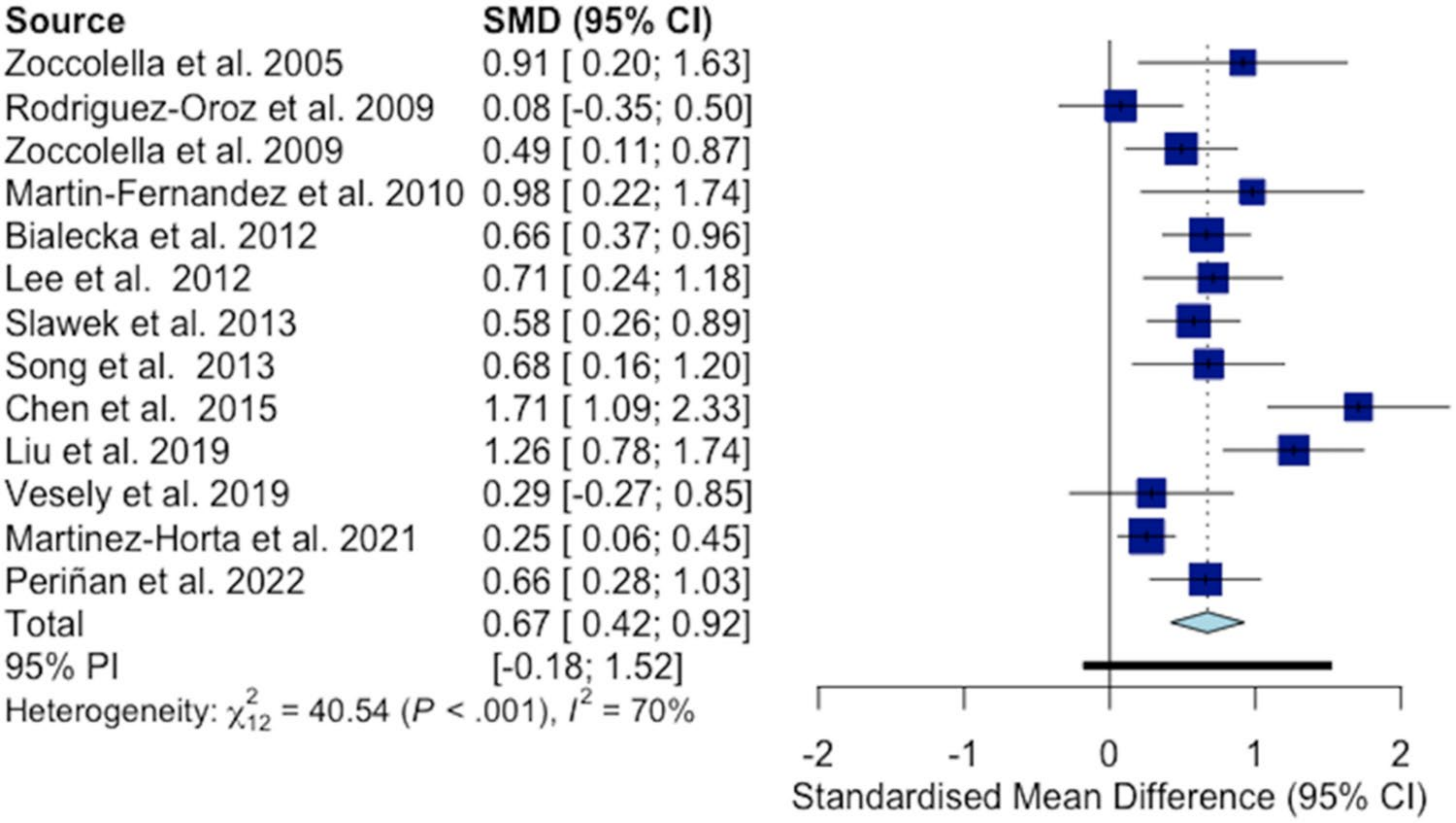
Forest plot displays random-effects meta-analysis results of the association between homocysteine levels and cognitive impairment in Parkinson’s disease. The overall standard mean difference between groups and its 95% confidence interval are represented by the light blue diamond

The standardized mean difference (SMD) for each individual study and the pooled effect size (ES) are shown in Fig. 1. PD patients with cognitive impairment had higher Hcy levels compared to PD patients without cognitive decline (SMD = 0.67; 95% CI 0.42–0.92, $\mathcal{X}$^2^_12_ = 40.54, *I*^2^ = 70.4%, *P* < 0.001).

A significant risk of publication bias was detected as demonstrated by the presence of asymmetries in the funnel plot and confirmed by the Egger’s test (*P* = 0.023) (Fig. S2). Sensitivity analysis showed that the Chen et al. study had a major influence on overall heterogeneity with little impact on the pooled ES, while the Martinez-Horta et al. study was the most influential on the pooled ES (Fig. S3). In the “leave-one-out” analysis, the pooled ES as well as the heterogeneity decreased when excluding the Chen et al. study (SMD = 0.59, 95% CI 0.38–0.79, *I*^2^ = 59%) (Fig. S4).

A subgroup analysis was performed to explore this heterogeneity. This analysis was based on the study design and showed no impact of this potential moderator on the pooled ES ($\mathcal{X}$^2^_1_ = 2.58, *P* = 0.110). The impact of potential continuous moderators (year of publication, age, percentage of men in the samples studied, folate, and vitamin B12 levels) on the pooled ES was assessed with meta-regression. This analysis did not produce a significant association between potential moderators and pooled ES (Table S2).

### Influence of genetic factors on biochemical parameters

We investigated whether the selected polymorphisms were associated with Hcy levels in PD and found an association between the *MTHFR* rs1801133 TT genotype and increased Hcy levels in PD after correction for multiple testing (TT vs. CC: *β* = 3.79; 95% CI 1.76–5.82; corrected *P* = 0.026) (Table 2). Furthermore, we assessed whether these polymorphisms influenced vitamin B12 levels. In this sense, no variants were significantly associated with vitamin B12 levels after adjusting for sex and age. Regarding folate levels, the *MTHFR* rs1801131 CC genotype was found to be significantly associated after correction for multiple testing (CC vs. AA: *β* = 3.03; 95% CI 1.38–4.69; corrected *P* = 0.031).

**Table 2:** Levels of homocysteine, vitamin B12 and folate corresponding to the different genotypes of the polymorphisms in PD patients

	Total PD											
	Hcy	*β* (95% CI)	*P* value^a^	Adjusted *P* value*	Vit B12	*β* (95% CI)	*P* value^b^	Adjusted *P* value*	Folate	*β* (95% CI)	*P* value^b^	Adjusted *P* value*
*MTHFR* rs1801133												
TT	20.6 ± 9.9	3.787 (1.758/5.816)	0.002	**0.026**	342.4 ± 142.7	− 41.441 (− 118.149/35.267)	0.373	1.000	7.5 ± 3.8	− 1.552 (− 3.080/− 0.024)	0.095	1.000
CT	17.8 ± 6.3	1.083 (− 0.257/2.422)	0.183	1.000	428.8 ± 251.0	44.185 (− 6.100/94.469)	0.148	1.000	8.1 ± 3.9	− 1.068 (− 2.070/− 0.067)	0.079	0.874
CC	17.1 ± 5.5	Ref.	Ref.	Ref.	384.0 ± 137.0	Ref.	Ref.	Ref.	9.2 ± 4.7	Ref.	Ref.	Ref.
TT vs. CT + CC	–	3.136 (0.922/5.349)	0.006	**0.030**	–	− 68.240 (− 152.000/15.490)	0.112	0.558	–	0.853 (− 2.576/0.768)	0.290	1.000
*MTHFR* rs1801131												
CC	18.1 ± 5.5	0.054 (− 2.277/2.386)	0.969	1.000	377.5 ± 115.5	− 22.336 (− 108.143/63.470)	0.668	1.000	11.2 ± 4.4	3.033 (1.376/4.689)	0.003	**0.031**
AC	17.9 ± 6.2	− 0.424 (− 1.766/0.919)	0.602	1.000	415.6 ± 275.0	18.236 (32.227/68.700)	0.551	1.000	8.4 ± 4.2	0.352 (− 0.622/1.326)	0.551	1.000
AA	17.8 ± 7.1	Ref.	Ref.	Ref.	398.3 ± 163.2	Ref.	Ref.	Ref.	8.0 ± 3.9	Ref.	Ref.	Ref.
CC vs. AC + AA	–	0.234 (− 2.443/2.911)	0.864	1.000	–	− 29.940 (− 128.500/68.610)	0.552	1.000	–	2.886 (0.983/4.788)	0.003	**0.016**
*COMT* rs4680												
AA	19.5 ± 9.1	1.213 (− 0.562/2.988)	0.260	1.000	389.9 ± 176.5	− 10.800 (− 74.551/52.952)	0.780	1.000	7.8 ± 4.2	− 0.581 (− 1.886/0.725)	0.464	1.000
AG	17.4 ± 6.2	− 0.106 (− 1.436/1.225)	0.896	1.000	402.1 ± 219.5	1.570 (− 46.228/49.369)	0.957	1.000	8.5 ± 4.2	0.237 (− 0.742/1.216)	0.690	1.000
GG	18.1 ± 6.0	Ref.	Ref.	Ref.	398.0 ± 183.2	Ref.	Ref.	Ref.	8.2 ± 4.2	Ref.	Ref.	Ref.
AA vs. AG + GG	–	1.275 (− 0.611/3.161)	0.187	0.932	–	− 11.74 (− 79.280/55.800)	0.734	1.000	–	− 0.722 (− 2.106/0.662)	0.308	1.000
*TCN2* rs1801198												
GG	16.2 ± 4.4	− 0.879 (− 2.667/0.910)	0.418	1.000	467.1 ± 201.4	61.488 (− 5.280/128.256)	0.130	1.000	8.3 ± 4.0	0.132 (− 1.213/1.476)	0.872	1.000
GC	18.1 ± 7.3	0.342 (− 1.025/1.709)	0.680	1.000	374.6 ± 188.6	− 30.005 (− 81.043/21.033)	0.333	1.000	8.7 ± 4.2	0.512 (− 0.516/1.539)	0.412	1.000
CC	18.3 ± 6.5	Ref.	Ref.	Ref.	406.4 ± 224.7	Ref.	Ref.	Ref.	8.3 ± 4.3	Ref.	Ref.	Ref.
GG vs. GC + CC	–	− 1.055 (− 3.001/0.890)	0.289	1.000	–	77.420 (5.032/149.800)	0.037	0.186	–	− 0.140 (− 1.596/1.317)	0.851	1.000
*MTRR* rs1801394												
GG	18.0 ± 6.0	− 0.208 (− 2.150/1.005)	0.860	1.000	370.9 ± 137.2	7.107 (− 53.024/67.238)	0.0748	0.823	7.6 ± 4.4	1.108 (− 0.351/2.567)	0.211	1.000
AG	17.6 ± 6.9	− 0.609 (− 2.223/1.005)	0.534	1.000	402.8 ± 194.3	Ref.	0.1607	1.000	8.7 ± 4.4	1.007 (− 0.207/2.221)	0.172	1.000
AA	18.4 ± 6.5	Ref.	Ref.	Ref.	450.9 ± 285.4	Ref.	Ref.	Ref.	8.7 ± 4.4	Ref.	Ref.	Ref.
GG vs. AG + AA	–	0.236 (− 1.595/2.068)	0.801	1.000	–	− 41.610 (− 110.600/27.430)	0.239	1.000	–	0.384 (− 1.006/1.775)	0.589	1.000

Significant *P* values are marked in bold

*PD* Parkinson’s disease, *Hcy* homocysteine, *Vit B12* vitamin B12, *LEDD* total levodopa equivalent daily dose, *CI* confidence interval, *Ref.* reference

^a^Linear regression model adjusted for sex, age, LEDD, folate and vitamin B12 levels

^b^Linear regression model adjusted for sex and age

*Bonferroni *P* value adjustment

### Comparison of PD patients with and without cognitive impairment

None of the SNPs were significantly associated with cognitive impairment in PD after correction for multiple tests (Table 3).

**Table 3:** Genetic association between the genetic polymorphisms and the development of cognitive impairment in PD

	OR (95% CI)	*P* value^a^	Adjusted *P* value*
*MTHFR* rs1801133			
TT	1.010 (0.893–1.143)	0.895	1.000
CT	0.956 (0.881–1.037)	0.364	1.000
CC	Ref.	Ref.	Ref.
*MTHFR* rs1801131			
CC	1.021 (0.888–1.174)	0.807	1.000
AC	1.030 (0.950–1.116)	0.544	1.000
AA	Ref.	Ref.	Ref.
*COMT* rs4680			
AA	0.925 (0.833–1.027)	0.221	1.000
AG	1.000 (0.926–1.081)	0.997	1.000
GG	Ref.	Ref.	Ref.
*TCN2* rs1801198			
GG	1.016 (0.912–1.132)	0.809	1.000
GC	1.030 (0.949–1.119)	0.552	1.000
CC	Ref.	Ref.	Ref.
*MTRR* rs1801394			
GG	0.940 (0.856–1.032)	0.877	1.000
AG	0.989 (0.884–1.107)	0.277	1.000
AA	Ref.	Ref.	Ref.

*OR* odds ratio, *CI* confidence interval, *Ref.* reference

^a^Logistic regression model adjusted for sex, age, LEDD, disease duration, folate and vitamin B12 levels

*Bonferroni *P* value adjustment

## Discussion

Cognitive decline in PD has been the subject of increasing research in recent decades. In our case–control study, PD patients with cognitive impairment showed increased Hcy levels compared to PD patients without cognitive impairment. The results of our meta-analysis supported the positive association between Hcy levels and cognition in PD. Furthermore, we demonstrated that the *MTHFR* rs1801133 TT genotype led to higher Hcy levels in PD patients, while the *MTHFR* rs1801131 CC genotype resulted in higher levels of folate concentration. However, the SNPs studied were not associated with cognitive impairment in PD.

Extensive clinical data support the role of hyperhomocysteinemia as a risk factor for cognitive impairment. Hcy can directly exert toxic effects on neurons by oxidative stress injury, DNA damage, and altering the expression of the NMDA receptor, leading to dysregulation in calcium homeostasis, mitochondrial function, neuronal autophagy, and apoptosis [30]. Additionally, Hcy can cause damage to vascular endothelial function and alter the permeability of the blood–brain barrier, resulting in small vessel disease in the brain [31]. Some studies have found that endothelial inflammation under high Hcy conditions promoted vascular injury, which, in turn, led to cognitive impairment [32].

The increased Hcy levels found in our cohort of PD patients with cognitive impairment are consistent with the data reported in the literature [10]. However, some relatively few studies failed to demonstrate this relationship [19, 20, 33]. The results of a study in a Spanish cohort found no evidence of an association between Hcy plasma levels and cognitive impairment and dementia in PD [20]. The differences seen with our study could be explained by the screening tests used for the assessment of cognitive status. Rodriguez-Oroz et al*.* evaluated the cognitive function with the MMSE, but also with the Blessed Dementia Rating Scale, which was not included in our cognitive evaluation battery for diagnosis of cognitive impairment. Annanmaki et al. found no correlation between Hcy levels and neuropsychological performance in a study cohort of 40 patients with PD [33]. Furthermore, Camicioli et al. showed that Hcy did not correlate with global cognition measures in a Canadian population of 51 patients with PD [19]. Notably, the studies failing to find a relationship involved relatively small sample sizes (*N* = 40 and *N* = 51, respectively), which would explain the inconsistencies seen with our study.

The meta-analysis performed confirmed that the Hcy levels were higher in the PD patients with cognitive impairment. The results of this meta-analysis may guide power analysis and sample size estimation for future observational studies as well as preclinical and clinical studies. One major limitation is that the substantial heterogeneity observed among all the included studies could not be clarified. Considering that some of the studies analyzed few PD-specific reported characteristics, we were unable to assess other factors that may have contributed to the observed heterogeneity. Some lifestyle factors such as dietary habits and physical exercise, might influence the metabolism of homocysteine. For this reason, future research should address the contribution of these factors to the observed heterogeneity [34, 35].

Among the genetic causes of hyperhomocysteinemia, the *MTHFR* gene is involved in the metabolism of Hcy and methionine, as well as in methylation processes. Although several polymorphisms have been described for this gene, the variant rs1801133 is the most frequently investigated due to its functional impact. Several studies have addressed the association between the *MTHFR* rs1801133 variant and increased Hcy levels in patients with PD. Our observations are in line with those of Bialecka et al*.*, in which the *MTHFR* rs1801133 variant was the genetic determinant of Hcy levels in patients with PD [11]. On the other hand, we also demonstrated the impact of the *MTHFR* rs1801131 variant on folate levels in patients with PD. To our knowledge, this is the first study to link *MTHFR* rs1801131 with folate levels in PD.

Relatively few studies have investigated the association between genetic factors involved in Hcy metabolism and cognitive dysfunction in PD patients [11, 20, 36]. To date, no significant association has been shown between cognitive impairment in PD and the *MTHFR* C677T, *MTHFR* A1298C, *TCN2* G776C, *SLC19A1* G80A, *COMT* G472A, *MTR* A2756G, and *CBS* 844ins68 polymorphisms. In agreement with these results, we failed to identify a relationship between the polymorphisms and cognitive impairment in PD.

Interestingly, a case–control study revealed that men from the Health in Men Study cohort with the *MTHFR* C677T TT genotype  had 46% higher odds of cognitive impairment than men with the CC genotype [37]. Furthermore, a recent meta-analysis has demonstrated the role of the *MTHFR* C677T polymorphism in Alzheimer’s disease in Asians but not in Caucasian populations [38]. Thus, it would be of interest to further study the role of the *MTHFR* C677T variant in larger populations of PD and, in addition, in populations with different ethnic backgrounds, to assess whether the *MTHFR* variants have considerable variation between different ethnic groups.

This study has some limitations. First, cognitive impairment was not assessed with the same standard cognitive scale during follow-up. However, all the scales used to assess cognitive performance were internationally accepted and successfully distinguished PD patients with and without cognitive impairment. Furthermore, we did not assess cognitive performance in a population of healthy controls, which may represent an important limitation since we could not elucidate whether the effect of Hcy on cognition in PD might interact in a different way than in healthy controls. Another limitation was the sample size of our case–control study, but the results of the meta-analysis reinforced the association described in the case–control study.

In conclusion, our case–control study demonstrated that increased Hcy levels were associated with cognitive decline in PD. The results of our meta-analysis further supported the positive association between Hcy levels and cognition in PD. No association was found between polymorphisms in genes implicated in Hcy metabolism and cognitive impairment in PD patients. Large-scale studies in ethnically diverse populations will be required to definitively determine the relationship between *MTHFR* and cognitive impairment in PD.

## Supplementary Information

Below is the link to the electronic supplementary material.

Supplementary file1 (DOCX 9448 KB)
