# Supplementary material for: Homocysteine levels, genetic background, and cognitive impairment in Parkinson’s disease
Source: J Neurol. 2022 Sep 28;270(1):477–85. doi: 10.1007/s00415-022-11361-y (PMC9813118; doi:10.1007/s00415-022-11361-y)
Supplement: Supplementary file 1 — Supplementary file1 (DOCX 9448 KB) [file 415_2022_11361_MOESM1_ESM.docx]

**SUPPLEMENTARY DATA**

**Meta-analysis**

1. **Methods**

- *Search strategy and eligibility criteria*

A comprehensive literature search was conducted in elec­tronic scientific databases (PubMed, Scopus, Ovid, Medline, Embase, Web of Science, and Cochrane) in February 2022 to identify relevant studies on the asso­ciation between Hcy levels and cognitive impairment in PD. We used the following search string: "Parkinson" OR “Parkinson’s Disease” AND "homocysteine” OR “hyperhomocysteinemia” AND “cognition” OR “cognitive impairment” OR “dementia”. Articles were included if the following inclusion criteria were met: (1) case-control studies describing the association between Hcy levels and cognitive impairment in PD; (2) studies had a cognitive normal PD group; and (3) studies had to include both means and standard deviations or sufficient statistical information (*t*, *F*, χ, Z-scores or p-values). No restrictions were applied regarding sample size or time of publication. To assess addition­al studies not indexed in common databases, all retrieved ref­erences of the enrolled studies and recent published review arti­cles were also checked.

- *Data extraction*

Two investigators (MTP and PGG) inde­pendently extracted the data. Discrepancies were resolved by a third investigator (DMG). We collected the following data: (1) general information: first author, publication year, study design, and cohort selected; (2) individual characteristics: sample size, age, and sex; and (3) mean and standard deviation of homocysteine, folate, and vitamin B12 levels. The corresponding author of the studies was contacted for further information when required.

- *Statistical analysis*

The "meta" package in R was used. Effect sizes were calculated as standardized mean differences of homocysteine levels between PD patients with and without cognitive impairment using the Hedges’ g statistic, which provides an unbiased effect size adjusted for sample size.

Between-study heterogeneity was assessed using the Cochrane Q test and I^2^ statistic. When heterogeneity was significant (p<0.05 or I^2^>50%), we used the random effects model to evaluate the overall effect size; otherwise, the fixed effects model was used. Publication bias was evaluated by a funnel plot and Egger’s test. Sensitivity analyses were undertaken to assess the influence of individual studies on the overall effect estimate. Subgroup and meta-regression analyses using mixed-effects models were conducted to detect the sources of heterogeneity.

1. **Results**

**Figure 1.** PRISMA flowchart of the literature search and study selection process.

Records identified through database searching (n = 723)

Additional records identified through other sources (n = 0)

Studies included in quantitative synthesis (meta-analysis)
(n = 12)

Full-text articles assessed for eligibility
(n = 29)

Records excluded with reason: not relevant (n = 309)

Records screened
(n = 338)

Identification

Elegibility

Included

Screening

Records after duplicates removed (n = 338)

Full-text articles excluded, with reasons (n = 17):
-not divided into PD with and without cognitive impairment groups (n = 14)
-no data available (n = 3)

**Table S1.** Main descriptive data of individual studies included in the meta-analysis.

| **Study name and year** | **PD without cognitive impairment** | | | | | | **PD with cognitive impairment** | | | | | |
| --- | --- | --- | --- | --- | --- | --- | --- | --- | --- | --- | --- | --- |
|  | **N** | **Hcy,**  **μmol/L**  **mean** ± **SD** | **Folate,**  **ng/mL**  **mean** ± **SD** | **Vit B12, pg/mL**  **mean** ± **SD** | **Age, Years**  **mean** ± **SD** | **Sex,**  **% men** | **N** | **Hcy,**  **μmol/L**  **mean** ± **SD** | **Folate,**  **ng/mL**  **mean** ± **SD** | **Vit B12, pg/mL**  **mean** ± **SD** | **Age, Years**  **mean** ± **SD** | **Sex,**  **% men** |
| Zoccolella et al. 2005 [17] | 21 | 15.80$\pm$4.40 | 6.0$\pm$3.3 | 476.00$\pm$178.00 | 63.70$\pm$7.00 | 71.4 | 14 | 21.20$\pm$7.40 | 5.5$\pm$3.80 | 454.00$\pm$308.00 | 67.80$\pm$5.80 | 71.4 |
| Rodriguez-Oroz et al. 2009 [20] | 37 | 14.90$\pm$4.70 | 10.20$\pm$5.60 | 400.10$\pm$222.20 | 69.97$\pm$6.50 | 54.6 | 52 | 15.27$\pm$4.93 | 10.91$\pm$5.40 | 368.78$\pm$177.27 | 72.91$\pm$5.75 | 63.4 |
| Zoccolella et al. 2009 [18] | 79 | 15.80$\pm$8.50 | 8.40$\pm$6.10 | 508.00$\pm$381.00 | 65.40$\pm$8.00 | 57.0 | 42 | 20.70$\pm$12.10 | 7.90$\pm$5.40 | 406.00$\pm$204.00 | 71.20$\pm$6.30 | 64.3 |
| Martin-Fernandez et al. 2010 [14] | 36 | 13.00$\pm$5.10 | 14.60$\pm$4.20 | 425.00$\pm$146.70 | 65.10$\pm$7.40 | NA | 9 | 18.20$\pm$5.70 | 12.30$\pm$5.20 | 342.20$\pm$132.80 | 72.20$\pm$3.80 | 55.56 |
| Lee et al. 2012 [12] | 38 | 11.46$\pm$3.44 | 15.21$\pm$11.34 | 686.55$\pm$323.69 | 64.66$\pm$7.64 | 42.1 | 35 | 23.97$\pm$24.93 | 13.01$\pm$12.01 | 658.22$\pm$439.46 | 72.46$\pm$7.00 | 37.1 |
| Bialecka et al. 2012 [11] | 153 | 16.50$\pm$6.30 | 9.30$\pm$4.20 | 347.00$\pm$147.00 | 62.10$\pm$9.70 | 51.0 | 64 | 21.30$\pm$9.00 | 8.50$\pm$4.40 | 304.00$\pm$141.00 | 68.80$\pm$9.30 | 53.1 |
| Slawek et al. 2013 [7] | 135 | 16.45±6.33 | 9.30±3.84 | 355.36±150.51 | 61.91±9.09 | 45.9 | 57 | 20.86±10.04 | 7.98±3.39 | 303.16±134.30 | 67.98±8.79 | 50.9 |
| Song et al. 2013 [15] | 33 | 11.59±3.60 | 15.21±11.34 | 641.92±298.90 | 66.45±6.60 | 42.4 | 28 | 18.80±15.00 | 13.01±12.01 | 581.24 ±359.93 | 70.50±6.75 | 42.9 |
| Chen et al. 2015 [16] | 22 | 14.12±1.64 | NA | NA | NA | NA | 36 | 18.28±2.76 | NA | NA | NA | 55.6 |
| Liu et al. 2019 [13] | 40 | 14.10±5.70 | 12.40±4.50 | 378.70±128.20 | 61.70±10.60 | 60.0 | 41 | 21.70±6.20 | 9.20±3.70 | 354.10±123.50 | 63.30±12.30 | 53.7 |
| Vesely et al. 2019 [39] | 40 | 12.40±3.00 | 7.1$\pm$2.5 | 308.50$\pm$127.10 | 62.80$\pm$7.90 | 62.5 | 18 | 13.30±3.30 | 7.80$\pm$5.10 | 291.00$\pm$91.30 | 66.80$\pm$8.20 | 50.0 |
| Martinez-Horta et al. 2021 [40] | 393 | 14.00±5.30 | NA | 396.40±152.20 | 60.80±8.80 | 62.4 | 140 | 15.47$\pm$7.01 | NA | 388.45$\pm$196.45 | 67.29$\pm$6.81 | 57.1 |
| Periñan et al. 2022 | 214 | 17.5$\pm$6.3 | 8.30$\pm$4.10 | 396.90$\pm$200.50 | 61.70$\pm$11.50 | 60.3 | 32 | 21.80$\pm$7.90 | 8.10$\pm$4.70 | 413.10$\pm$200.50 | 69.30$\pm$7.80 | 46.9 |

PD-CN, Parkinson’s disease cognitive normal; PD-CI, Parkinson’s disease with cognitive impairment; N, number of subjects; y, years; N/A, non-available; Vit B12, vitamin B12.

**Figure S2**. Funnel plot. Evident asymmetry was observed as demonstrated by the presence of asymmetries in the funnel plot and confirmed by Egger’s test (p=0.023).

**
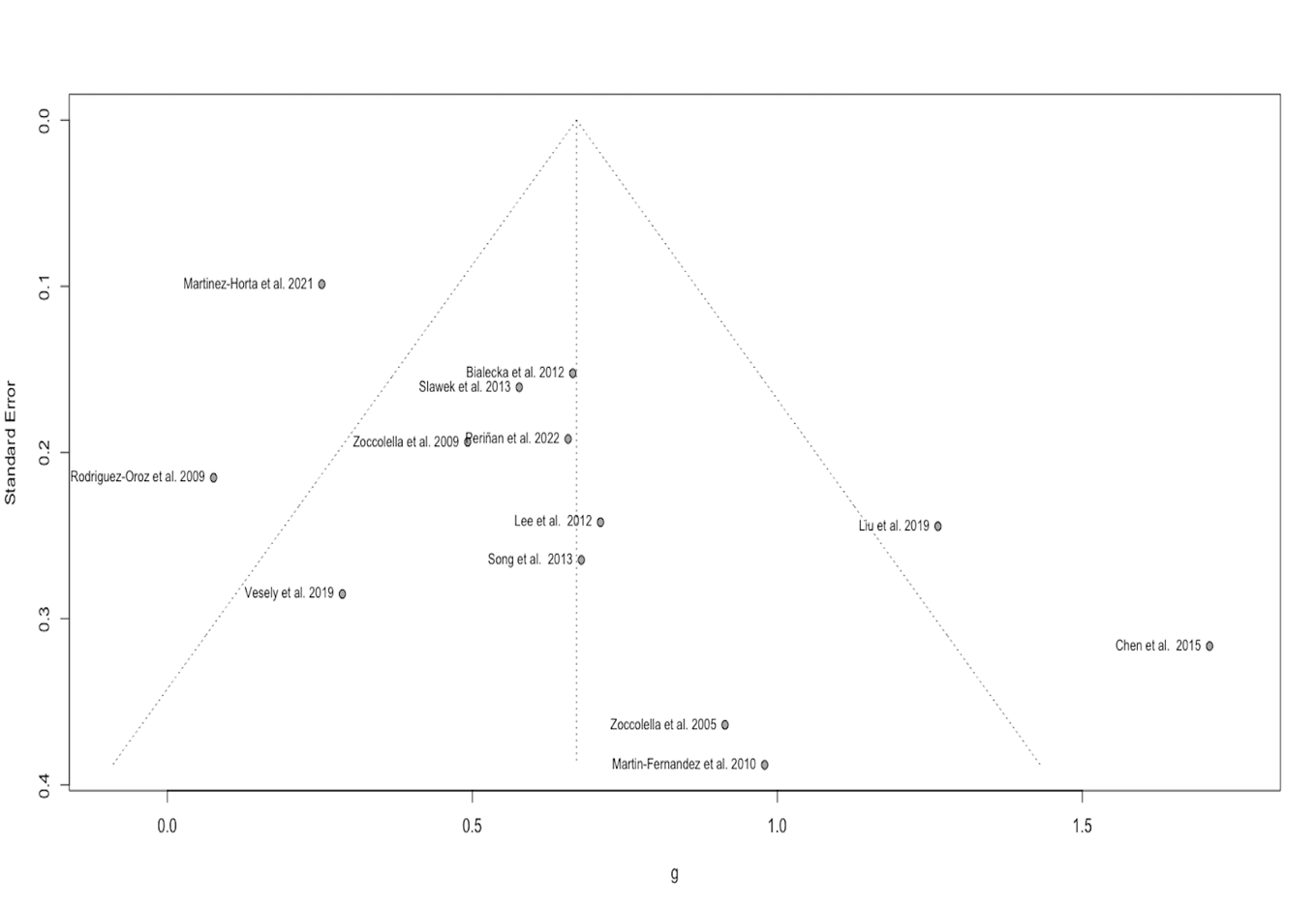
**

**Figure S3.** Baujat Plot graphic. The contribution of each study to the overall heterogeneity was reflected on the horizontal axis, and its influence on the pooled effect size on the vertical axis. The Baujat Plot showed that the Chen et al study had a major influence on the global heterogeneity with little impact on the global effect size, whereas the Martinez-Horta et al was the most influential on the global effect size.

**
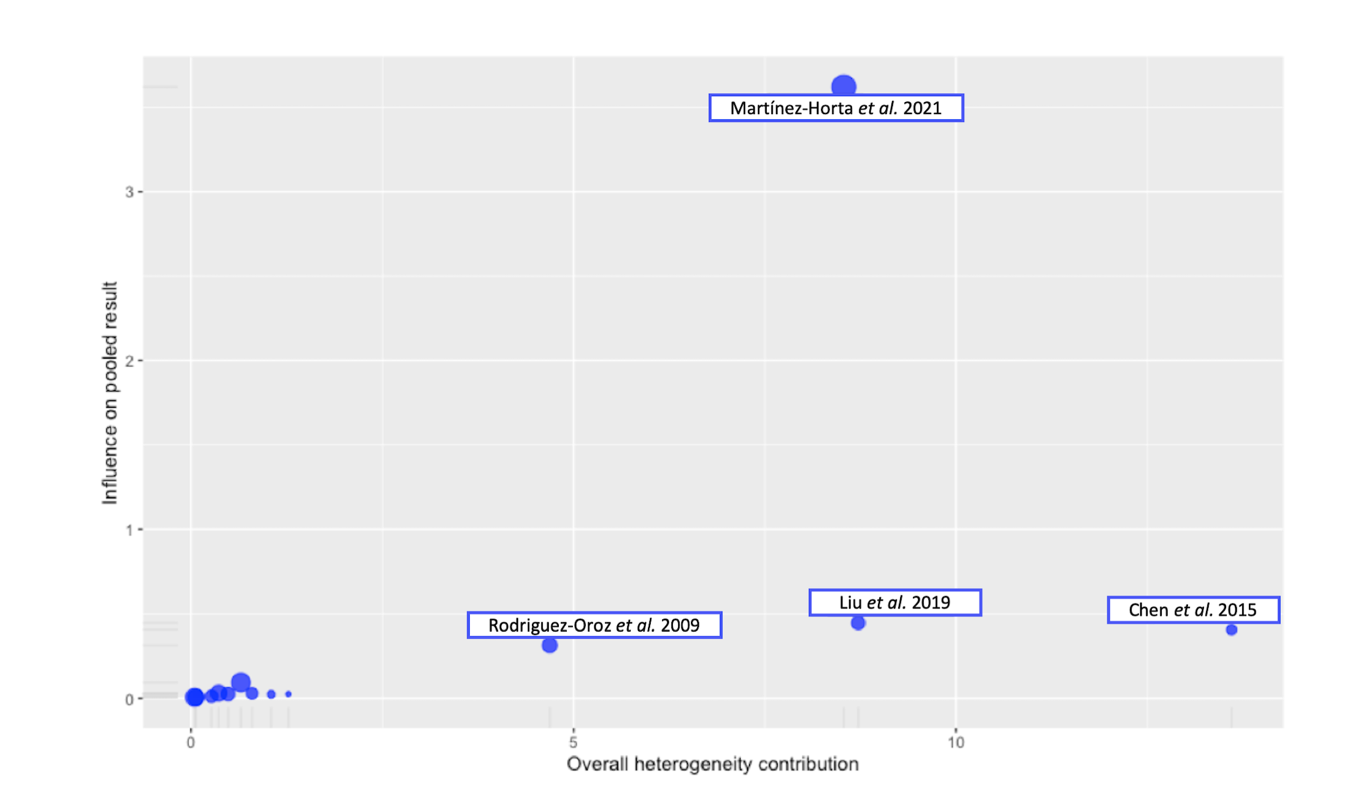
**

**Figure S4.** Forest plot excluding Chen et al study. When the meta-analysis was repeated excluding the Chen et al study, the overall effect size remained the same and the heterogeneity did not substantially decrease.


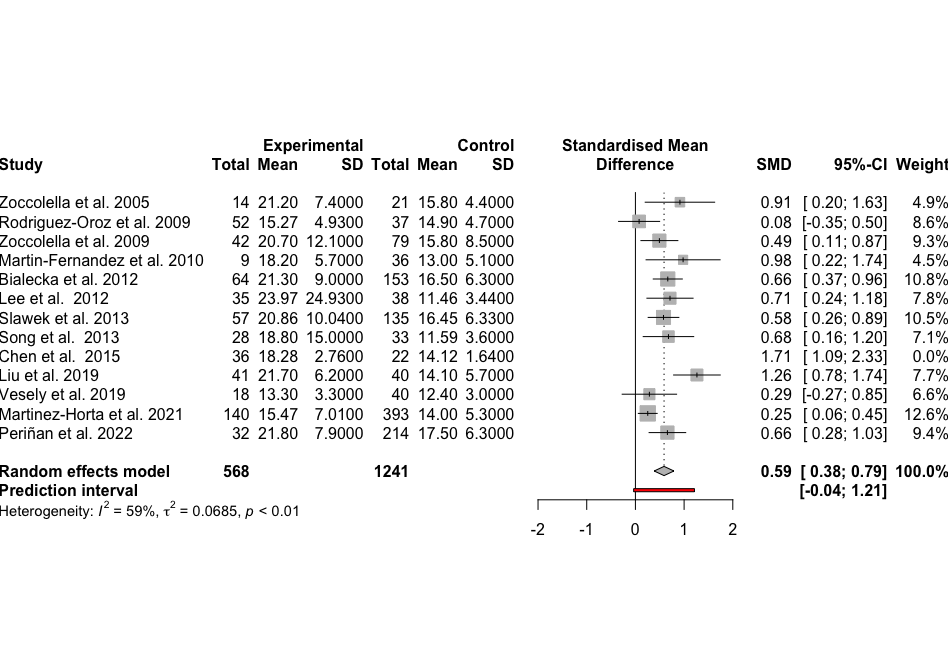


**Table S2**. Meta-regression analyses: no heterogeneity was identified among studies.

|  | **% Heterogeneity** | **β** | **P value** |
| --- | --- | --- | --- |
| Year of publication | 0.00% | -0.001 | 0.986 |
| Age of PD patients with cognitive impairment | 5.08% | -0.043 | 0.241 |
| Sex of PD patients with cognitive impairment | 0.00% | -0.004 | 0.774 |
| Folate levels of PD patients with cognitive impairment | 0.00% | -0.003 | 0.957 |
| Vit B12 levels of PD patients with cognitive impairment | 0.00% | 0.0002 | 0.779 |

PD, Parkinson’s disease; Vit B12, vitamin B12.
